# Editorial: New vaccines and drugs for human microbial infections

**DOI:** 10.3389/fmicb.2026.1837270

**Published:** 2026-04-09

**Authors:** Pedro José Alcolea, Ana Alonso, Thamir A. Alandijany

**Affiliations:** 1 Biological Research Centre Margarita Salas, Spanish Research Council (CIB-CSIC), Madrid, Spain; 2 Special Infectious Agents Unit BSL-3, King Fahd Medical Research Center, King Abdulaziz University, Jeddah, Saudi Arabia; 3 Department of Medical Laboratory Sciences, Faculty of Applied Medical Sciences, King Abdulaziz University, Jeddah, Saudi Arabia

**Keywords:** bacteria, drugs, fungi, immune response and pathology, infections, pharmacology, vaccines, viruses

The primary objective of this Research Topic was to report on newly confirmed drugs and vaccines and their implementation for human microbial infections. Furthermore, it aimed to describe innovative strategies for the discovery of novel candidates. Three Review and six Original Research articles are issued in this volume.

Two of the Reviews, the first being systematic, compile the state-of-the-art on vaccine development against a bacterial and a viral infection, respectively, whereas the third one focuses on topical treatment formulations to combat multidrug-resistant (MDR) pathogenic bacteria. In their Systematic Review, Taha et al. present a meta-analysis of five randomized controlled clinical trials that evaluated the safety and immunogenicity of the Vi-DT vaccine, a diphtheria toxoid-conjugated typhoid vaccine, in children under 2 years old. The vaccine showed a robust safety profile with just quickly resolved mild to moderate adverse events and comparable seroconversion to the WHO-prequalified Vi-TT vaccine, as well as a stronger immune memory response. Vi-DT is a safe, effective, and potentially cost-beneficial prophylactic tool against typhoid fever, caused by *Salmonella typhi*, a public health threat particularly associated with poor sanitation conditions. Shaikh et al. reviewed the current panorama on Chikungunya virus (CHIKV) vaccines. The CHIKV disease is a global health threat whose clinical manifestations range from acute symptoms such as fever, rash, and arthralgia to chronic rheumatism. CHIKV poses a serious risk to vulnerable populations like infants and the elderly, leading to fatal neuropathy. Effective viral clearance requires coordination between the innate and the adaptive immune responses. Most vaccines focus on the envelope E2 glycoprotein because neutralizing antibodies mainly target this antigen. Novel vaccines targeting multiple viral lineages, conceived in different formats, including mRNA, DNA, virus-like particles, and live-attenuated vaccines, are currently under development to improve the efficacy provided by the FDA-approved IXCHIQ vaccine. The review by Yang et al. provided a comprehensive overview of emerging strategies to combat MDR bacteria in cutaneous wound infections. Their review highlights innovative delivery platforms, such as hydrogels, liposomes, electrospun fibers, nanoparticles, and nanoemulsions, designed to enhance the therapeutic performance of bacteriophages and lysins. These approaches have shown promising potential in reducing bacterial burden and promoting wound healing.

All other contributions to this Research Topic are Original Research articles, one about vaccine development to prevent a digestive tract bacterial infection, two proposing general treatment screening strategies providing proof-of-concept examples of bacterial infection treatment, and three developing specific treatment strategies. Two of the latter are conceived for fungal infections and one for a bacterial pathogen.

Ruamsap et al. evaluated the safety, immunogenicity, and efficacy of several live-attenuated vaccine candidates against *Shigella dysenteriae* serotype 1 (Sd1) in three intragastrically-administered doses using a Rhesus macaque model closely mimicking the human disease. Shigellosis is a worldwide-distributed cause of morbidity and mortality in children, mainly in Africa and Asia, urgently requiring vaccines, but a major hurdle is the lack of appropriate animal models other than non-human primates. The authors focused on engineering the original WRSd1 strain to restore the accidentally-deleted fumarate nitrate reductase (*fnr*) gene (WRSd2 and WRSd4) and alternative versions featuring deletions in enterotoxin genes and lipid modifications (WRSd3 and WRSd5). The experimental challenge with the wild-type Sd1 1617 strain was performed 1 month after completion of the vaccination schedule. The candidates showed mild (WRSd2 and WRSd4) to no (WRSd3 and WRSd5) adverse events and significant protection against clinical shigellosis in all cases compared to the control group, with the beneficial effects of the *fnr* gene restoration for the host's immune response.

Shen et al. proposed a general methodological framework implementing a target-based drug development strategy exemplified with the evaluation of the therapeutic potential of licochalcone A (LAA) against pneumonia caused by methicillin-resistant *Staphylococcus aureus* (MRSA). By integrating computational network pharmacology with *in vivo* validation, the study identifies specific molecular targets, demonstrating that LAA mitigates lung injury and suppresses the proinflammatory cytokine storm (IL-1β, IL-6, TNF-α) through the inhibition of the NF-κB, MAPK (JNK/p38), and NLRP3 inflammasome pathways. The compound is a promising candidate for systematic treatment due to its antibacterial and anti-inflammatory efficacy and its favorable safety profile in mouse models. Another general strategy exemplified by a specific case is the standardized workflow combining classical ethnopharmacology-based drug discovery with computational modeling was applied by Sulieman et al. to investigate the medicinal plant *Rumex vesicarius* (Polygonaceae), a species adapted to arid environments in Saudi Arabia. The plant extract exhibited inhibitory activity against the growth and biofilm formation of *Bacillus subtilis* and *Pseudomonas aeruginosa*. GC–MS analysis identified 16 compounds, six of which showed potential binding affinity to the FtsZ protein, with D-amygdalin among the most prominent candidates.

Two different treatment strategies against fungal infections have been presented in this volume. Chen et al. identified an antifungal peptide from the WHIS1 protein, a member of the single domain von Willebrand factor type C (SVWC) family whose origin is the housefly, *Musca domestica* (Diptera: Muscidae). The capacity of some SVWC family members to protect certain insect species that produce them against fungal infections has been well studied. The novelty of this work relies on the discovered peptide featured to impair the capacity of *Candida albicans* to invade human tumor cells from the A549 and HeLa lines *in vitro*, leading to a decreased intracellular fungal burden and limited expression of virulence factors, namely all key hyphal formation factors, compared to the infection control. To improve colletochlorin B treatment of *Cryptococcus neoformans* infection, an opportunistic fungal pathogen able to cause life-threatening meningitis primarily affecting immunocompromised patients, Edrich et al. modified the chemical structure of this natural product by replacing chloride with bromide and the geranyl chain with alkyl triphenylphosphonium salt chains of various lengths in the orsellinaldehyde ring. As a result, these novel compounds targeting a mitochondrial alternative oxidase (AOX) were able to provide stronger inhibition values, thus leading to a more powerful disruption of the mitochondrial membrane potential and oxygen consumption.

Lee et al. evaluated parenteral administration of nemonoxacin, a next-generation broad-spectrum non-fluorinated quinolone against Gram-positive bacteria, to treat *Clostridioides difficile* infections (CDI). While oral vancomycin and fidaxomicin are currently being used for CDI treatment, patients with fulminant disease or severe ileus require effective non-oral alternatives. This study revealed that nemonoxacin has similar inhibitory effects against *C. difficile* to vancomycin *in vitro* and in fecal suspension models. Intraperitoneal injection of nemonoxacin alone or in combination with vancomycin significantly improved the survival rates and reduced mucosal damage with a favorable safety profile, yielding comparable results to current treatment strategies. The clinical relevance of this study is the possibility of implementing intraperitoneal treatment with nemonoxacin in nil by mouth patients.

In conclusion, these nine studies fulfill the aims of these Research Topic, spanning from the discovery to the review of novel treatment and vaccine strategies. To continue addressing these global health challenges, a second volume of this topic will be shortly issued, providing a continued platform for cutting-edge research in the field.

